# Mammalian Cell Surface Display as a Novel Method for Developing Engineered Lectins with Novel Characteristics

**DOI:** 10.3390/biom5031540

**Published:** 2015-07-20

**Authors:** Keisuke Soga, Hirohito Abo, Sheng-Ying Qin, Takuya Kyoutou, Keiko Hiemori, Hiroaki Tateno, Naoki Matsumoto, Jun Hirabayashi, Kazuo Yamamoto

**Affiliations:** 1 Department of Integrated Biosciences, Graduate School of Frontier Sciences, the University of Tokyo, Chiba 277-8562, Japan; E-Mails: keisuke.soga@gmail.com (K.S.); 137303@ib.k.u-tokyo.ac.jp (H.A.); qinshengying@gmail.com (S.-Y.Q.); kyoutou.takuya@sysmex.co.jp (T.K.); nmatsu@k.u-tokyo.ac.jp (N.M.); 2 Research Center for Stem Cell Engineering, National Institute of Advanced Industrial Science and Technology (AIST), Ibaraki 305-8568, Japan; E-Mails: keiko-hiemori@aist.go.jp (K.H.); h-tateno@aist.go.jp (H.T.); jun-hirabayashi@aist.go.jp (J.H.)

**Keywords:** leguminous lectin, cell surface display, carbohydrate-binding specificity, molecular engineering, scaffold

## Abstract

Leguminous lectins have a conserved carbohydrate recognition site comprising four loops (A–D). Here, we randomly mutated the sequence and length of loops C and D of peanut agglutinin (PNA) and expressed the proteins on the surface of mouse green fluorescent protein (GFP)-reporter cells. Flow cytometry, limiting dilution, and cDNA cloning were used to screen for several mutated PNAs with distinct properties. The mutated PNA clones obtained using NeuAcα2-6(Galβ1-3)GalNAc as a ligand showed preference for NeuAcα2-6(Galβ1-3)GalNAc rather than non-sialylated Galβ1-3GlcNAc, whereas wild-type PNA binds to Galβ1-3GlcNAc but not sialylated Galβ1-3GalNAc. Sequence analyses revealed that for all of the glycan-reactive mutated PNA clones, (i) loop C was eight amino acids in length, (ii) loop D was identical to that of wild-type PNA, (iii) residue 127 was asparagine, (iv) residue 125 was tryptophan, and (v) residue 130 was hydrophobic tyrosine, phenylalanine, or histidine. The sugar-binding ability of wild-type PNA was increased nine-fold when Tyr125 was mutated to tryptophan, and that of mutated clone C was increased more than 30-fold after His130 was changed to tyrosine. These results provide an insight into the relationship between the amino acid sequences of the carbohydrate recognition site and sugar-binding abilities of leguminous lectins.

## 1. Introduction

Numerous types of glycans are displayed on the surface of mammalian cells and glycans play various important roles in the biological functions of the cell. Profiling of glycan structures, especially on the cell surface, has been performed via either mass spectrometric analyses of released oligosaccharides [1] or lectin microarray analyses of the cell itself [2,3,4,5]. Although both methods are highly sensitive and reproducible, mass spectrometric analyses are associated with difficulties in quantitative determinations and specifying anomer structures, whereas lectin microarrays are unable to accommodate some glycan structures. A number of plant lectins, especially leguminous lectins, were studied extensively some decades ago. Leguminous lectins, which form the largest family of lectin proteins, consist of four subunits; each subunit has a carbohydrate recognition site that comprises four loops (A,B,C and D), and calcium and manganese ions are necessary for their sugar-binding activities [6]. Although the amino acid sequences and tertiary structures of various leguminous lectins are similar, they show a variety of sugar-binding specificities. Recognition of complex glycan structures by the sugar-binding site promotes the interaction of lectins with the elongated sugar structure via hydrogen bonds and van der Waals forces. The binding specificity of leguminous lectins is determined by residues located both within and close to the core region of the sugar-binding site. In previous studies, we identified the sugar-binding peptides of several leguminous lectins by affinity chromatography analyses of trypsin or lysylendopeptidase digests on haptenic sugar-immobilized columns [7,8,9]. Notably, all of these sugar-binding peptides were derived from loop C of the sugar-binding site, and the replacement of loop C of the galactose-binding *Bauhinia purpurea* lectin with that from the mannose-binding *Lens culinaris* lectin altered the binding specificity from galactose to mannose [10]. X-ray crystallographic analyses of several leguminous lectins in complex with sugar ligands indicated that the long loop C region is largely involved in both the interaction of a leguminous lectin with its glycan ligand and the determination of sugar-binding specificity.

Lectin engineering technology has recently advanced using site-directed mutagenesis, site-directed saturation mutagenesis, random mutagenesis, and DNA shuffling [11]. The engineered lectins having novel specificity could be useful probes since cell surface glycans on tumor cells are structurally changed in comparison to those of normal cells, and specific probes for such tumor-specific glycans could be anti-cancer drugs [12,13].

Here, we established a method for the efficient expression of leguminous lectin subunits on the surface of mammalian cells without the loss of sugar-binding ability. To evaluate the amino acid sequence, especially in its sugar-binding loops and the sugar-binding specificity of lectins, we introduced random mutations in sugar-binding loops of peanut agglutinin (PNA), expressed on the surface of mammalian cells, and successfully screened for mutated PNAs with novel sugar-binding specificities. The data demonstrated that sugar-binding loop C was largely involved in the sugar-binding specificity of the lectin and critical amino acid residues associated with sugar-binding activity and specificity were clarified.

## 2. Results

### 2.1. Construction of Mutated PNA Library Plasmids

**Figure 1:**
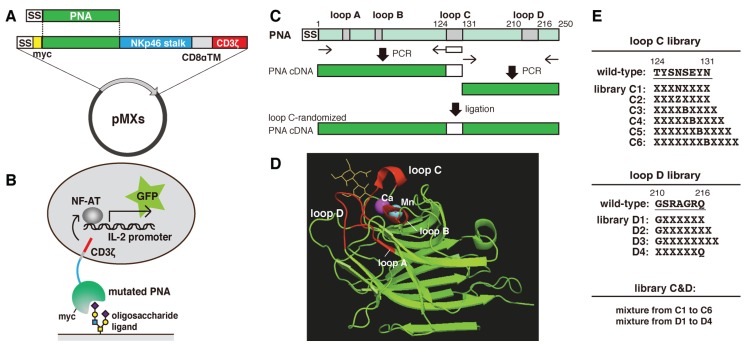
Overview of the methods used to prepare and analyze the mutated PNA libraries. (**A**) A schematic illustration of the pMXs vector used to express myc-tagged PNA fused to the natural killer cell p46-related protein stalk domain, CD8α transmembrane domain, and CD3ζ. SS, CD8β signal sequence; (**B**) A schematic illustration of the cell surface display method and green fluorescent protein (GFP)-reporter assay used to characterize the mutated PNA proteins; (**C**) A schematic illustration of the PCR-based method used to construct PNA libraries containing mutations in loop C of carbohydrate recognition site using degenerate oligonucleotides (white box); (**D**) Three-dimensional structure of PNA co-crystallized with *N*-acetyllactosamine (yellow) (1ciw). Carbohydrate recognition site consists of four loops (red) and calcium and manganese ions stabilize loop C for enhancing the interaction between the loop and a sugar ligand; (**E**) The sequences that were randomly mutated (X) in the loop C, loop D, and loop C&D PNA libraries (where B = Asp or Asn, and Z = Glu or Gln).

The carbohydrate recognition sites of various leguminous lectins, which consist of four peptide loops named A,B,C and D [14], share structural similarities. Loop C is the important contributor to the sugar-binding specificity [10,15], and it has been suggested that the length of loop D also contributes to this feature [16,17] (Figure 1D). Here, we attempted to alter the sugar-binding specificity of PNA by randomly mutating and extending the regions of the PNA cDNA encoding loop C and/or loop D (Figure 1C). To express PNA on the surface of reporter cells, pMXs vectors expressing myc-tagged PNA/CD3ζ fusion proteins (pMXs-PNA-CD3ζ) were constructed. Using this method, the following PNA-mutated library plasmids were prepared: six loop C mutants (C1–C6), four loop D mutants (D1–D4), and one loop C&D mutant (Figure 1E). In the C1 mutant, with the exception of Asn127, the remaining seven amino acid residues in loop C were substituted with random amino acids. The C2 mutant was similar to C1, with the exception that Asn127 was also substituted with Asp, Glu, or Gln. The C3, C4, C5, and C6 mutants were similar to C2 but contained an extension of one, two, three, and four amino acids, respectively. In the D1–D4 mutants, six of the seven amino acids in loop D were randomly mutated by polymerase chain reaction (PCR) (Figure 1E). Moreover, the D2 and D3 mutants contained an extension of one and two residues, respectively. The C&D mutant library was constructed by ligation of the loop C cDNAs from the mixed loop C libraries (C1–6) into the mixed loop D library plasmids (D1–4). All of the mutated PNA cDNA libraries were inserted into the pMXs retroviral expression vector (Figure 1A). The diversity (independent clone numbers) of each library is shown in Table 1.

**Table 1:** Diversity of each PNA library.

Library Name	Colony-Forming Unit
C1	4.0 × 10^6^
C2	3.0 × 10^6^
C3	1.8 × 10^6^
C4	1.6 × 10^6^
C5	1.5 × 10^6^
C6	2.0 × 10^6^
D1	1.4 × 10^5^
D2	1.1 × 10^5^
D3	1.0 × 10^5^
D4	3.3 × 10^5^
C&D	5.0 × 10^6^
Total	1.96 × 10^7^

### 2.2. Generation of 2B4 Reporter Cells Displaying Mutated PNAs on the Cell Surface

**Figure 2:**
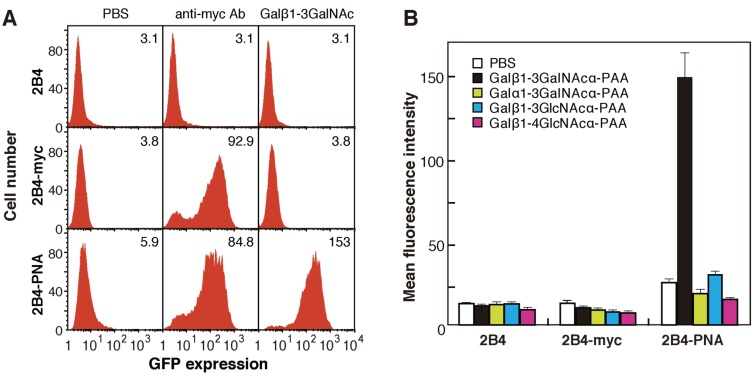
Reporter assays of PNA-expressing 2B4 cells. (**A**) Fluorescence-activated cell sorting (FACS) analyses of GFP expression in myc-tagged PNA-expressing (2B4-PNA), myc-tag-expressing (2B4-myc), and untransfected 2B4 cells grown on wells coated with anti-myc antibody (middle panels), Galβ1-3GalNAc-PAA (right panels), or PBS as a control (left panels). The numbers in each panel indicate the mean fluorescence intensity; (**B**) Reporter assay of GFP expression in 2B4-PNA cells grown on wells coated with several disaccharide-PAAs. The data are represented as the mean ± SD of n = 3 independent experiments.

The plasmids encoding the mutated PNA libraries were expanded in *Escherichia coli* and used to screen for novel lectins with distinct characteristics, including sugar-binding specificity. The pMXs retroviral expression vector was used to express mutated PNAs on the surface of mammalian cells; the use of this vector enabled efficient gene transfer and the stable expression of only one or a few kinds of cDNA per cell. The pMXs constructs were introduced into Plat-E cells to obtain retroviral particles containing mutated PNA cDNA. The retroviral particles were then transformed into 2B4 cells having a GFP transgene under the control of NF-AT. In transformed 2B4 cells expressing mutated myc-tagged PNA/CD3ζ fusion proteins on the cell surface, binding of PNA to an oligosaccharide ligand was transduced into intracellular GFP expression (Figure 1B). As controls, cells expressing wild-type PNA/CD3ζ (2B4-PNA) and myc-tag/CD3ζ fusion proteins (2B4-myc) were generated; culture of both of these kind of cells in wells coated with anti-myc antibody for 12 h induced GFP expression (Figure 2A). Culture of 2B4-PNA cells in Galβ1-3GalNAc-PAA-coated wells, but not Galα1-3GalNAc-PAA-, Galβ1-3GlcNAc-PAA-, or Galβ1-4GlcNAc-PAA-coated wells, induced GFP expression (Figure 2B). Because Galβ1-3GalNAc is a well-known ligand of PNA [18,19], these results indicate that exogenous PNA displayed on the surface of 2B4 cells was capable of transducing a signal into the cells via cross-linking induced by an immobilized sugar ligand. These data also demonstrate that the interaction between immobilized sugars and lectins displayed on 2B4 cell surfaces could be detected based on the intracellular GFP expression level.

### 2.3. Screening for Mutated Lectins with Novel Sugar-Binding Specificities

**Figure 3:**
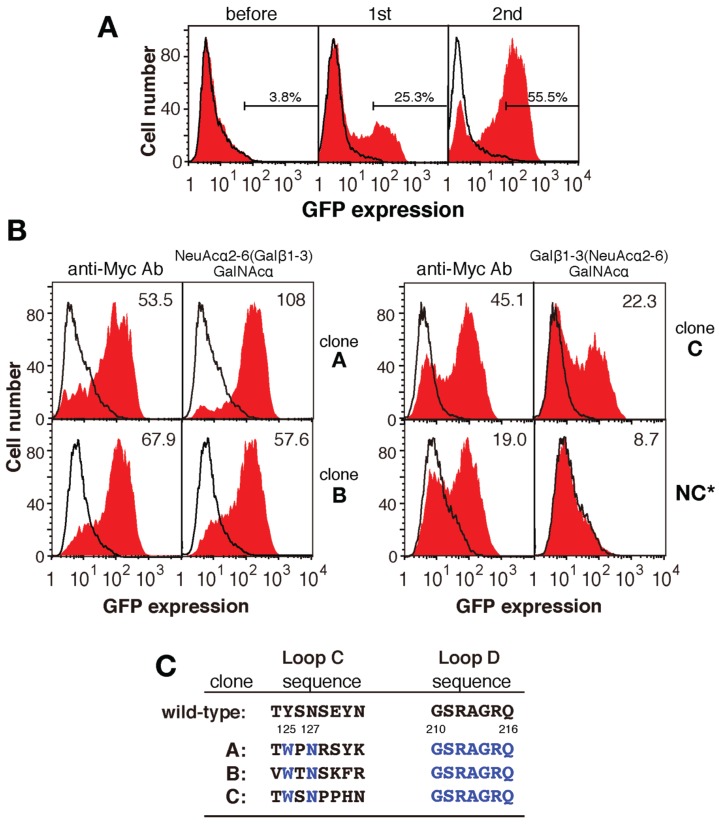
Enrichment of cells expressing mutated PNA. (**A**) Flow cytometric analyses of cells expressing mutated PNA before and after one or two rounds of selection. The red histogram and solid line indicate GFP expression in the presence or absence of NeuAcα2-6(Galβ1-3)GalNAc-PAA, respectively; (**B**) Flow cytometric analyses of three mutated PNA clones (A to C) obtained using an anti-myc antibody (left panels) or NeuAcα2-6(Galβ1-3)GalNAc-PAA-coated wells (right panels). Clone NC is representative of a negative clone. The red histogram and black line indicate GFP expression in the presence or absence of the ligand, respectively. The numbers in each panel indicate the mean fluorescence intensity; (**C**) The amino acid sequences of loops C and D of mutated PNA clones A–C (deduced from the nucleotide sequences). Blue letters indicate conserved amino acid residues.

**Figure 4:**
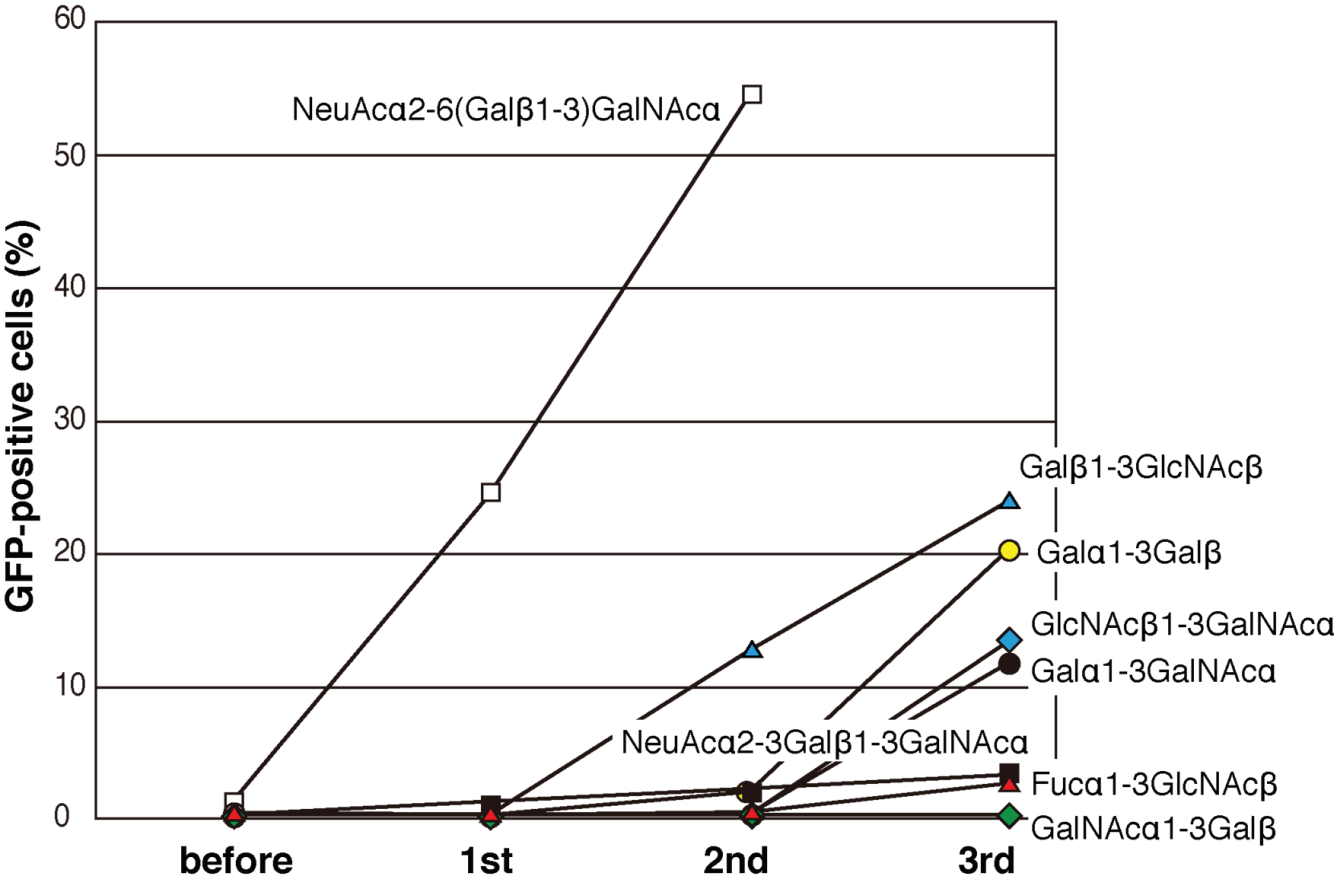
The efficiency of enrichment of GFP-positive 2B4 cells expressing mutated PNA using flow cytometry. GFP-positive cells were defined as cells that showed GFP expression higher than approximately 3% of the mutated PNA-expressing 2B4 cells before selection. The enrichment of GFP-positive mutated PNA-expressing 2B4 cells in wells coated with Manα-PAA, GlcNAcβ-PAA, Fucα-PAA, Fucα1-4GlcNAcβ-PAA, and Fucα1-3(Galβ1-4)GlcNAcβ-PAA was similar to that in wells coated with GalNAcα1-3Galβ-PAA.

Plasmids containing the mutated PNA libraries (C1–C6, D1–D4, and C&D) were expressed in 2B4 cells, which were cultured in wells coated with sugar-PAA derivatives for 12 h. Initially, NeuAcα2-6(Galβ1-3)GalNAcα-PAA and NeuAcα2-3Galβ1-3GalNAcα-PAA were used as ligands. Cell fractions expressing large amounts of GFP were collected by cell sorting (approximately 3% of the cell population in the first round), and the sorting process was repeated two-to-four times using each GFP-positive cell fraction. Compared with those cultured in uncoated wells, GFP-positive cells cultured in NeuAcα2-6(Galβ1-3)GalNAcα-PAA-coated wells were efficiently enriched after two rounds of sorting (Figure 3A and Figure 4 white squares). By contrast, after growth in NeuAcα2-3Galβ1-3GalNAcα-PAA-coated wells, GFP-positive cells were not effectively enriched even after three rounds of sorting (Figure 4 black squares). The same experiments were performed using 12 other sugar-PAAs as ligands, including terminally galactosylated, fucosylated, *N*-acetylglucosaminylated, and *N*-acetylgalactosaminylated oligosaccharides. Moderate enrichment of GFP-positive cells was achieved when Galβ1-3GlcNAcβ-PAA, Galα1-3Galβ-PAA, Galα1-3GalNAcα-PAA, and GlcNAcβ1-3GalNAcα-PAA were used as ligands (Figure 4). By contrast, GFP-positive cells were not enriched after three rounds of screening using Fucα-PAA, Fucα1-3GlcNAcβ-PAA, Fucα1-4GlcNAcβ-PAA, Fucα1-3(Galβ1-4)GlcNAcβ-PAA, Manα-PAA, GlcNAcβ-PAA, GalNAcα-PAA, or GalNAcα1-3Galβ-PAA as ligands (Figure 4B). These results suggest that a scaffold of βGal-binding PNA may be suitable for the recognition of terminal β-galactose residues.

**Figure 5:**
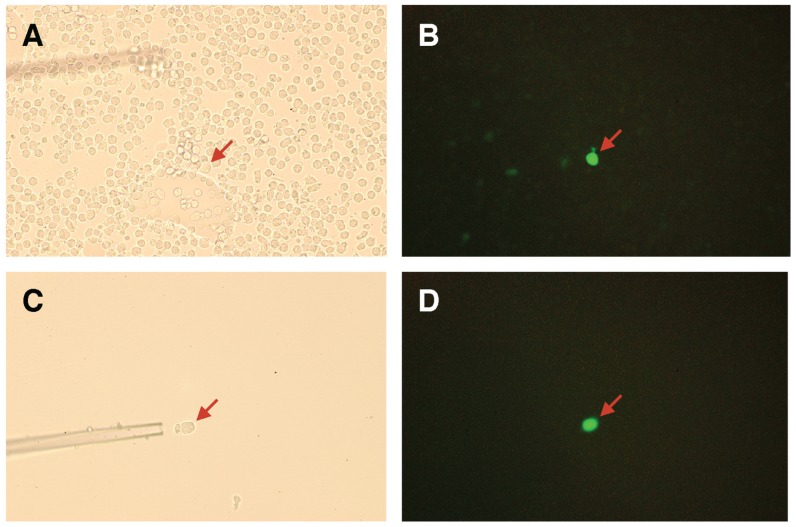
Expression of GFP in mutated PNA-expressing 2B4 cells. Mutated PNA library-expressing 2B4 cells were cultured in a NeuAcα2-6(Galβ1-3)GalNAcα-PAA-coated well (**A**,**B**). A GFP-positive cell was selectively manipulated using a glass capillary (**C**,**D**). GFP-positive cells (red arrow) were visualized using bright-field (**A**,**C**) or fluorescence (**B**,**D**) microscopes.

Next, GFP-positive cells obtained from two rounds of cultures in NeuAcα2-6(Galβ1-3)GalNAcα-PAA-coated wells were cloned by limiting dilution, and 15 independent cell clones expressing mutated PNA proteins were established. Alternatively, GFP-positive cells were collected using a combination of fluorescence microscopy and manipulator as shown in Figure 5. In case of manipulation method, enrichment of GFP-positive cells is not requisite for screening and had an advantage in rapid screening in comparison with the above method using flow cytometry. In this method, we collected more than twenty GFP-positive cells in total and then subjected them to the following genomic DNA extraction.

Genomic DNA was purified from each clone and the mutated PNA cDNAs were amplified by PCR. Overall, two-to-four different mutated PNA cDNAs were amplified from each GFP-positive cell clone, and 15 cDNAs were identified. Each cDNA was then subcloned into the pMXs-PNA-CD3ζ vector (replacing the wild-type PNA cDNA) and expressed in 2B4 cells. Each mutated PNA-expressing 2B4 cell was grown in NeuAcα2-6(Galβ1-3)GalNAcα-PAA-coated wells, and three positive clones (named A to C) were identified by flow cytometry (Figure 3B). The amino acid sequences of loops C and D in each mutated PNA protein are shown in Figure 3C.

### 2.4. Identification of the Sugar-Binding Specificities of Mutated PNA-IgG Fc Fusion Proteins Using a Glycan Microarray

**Figure 6:**
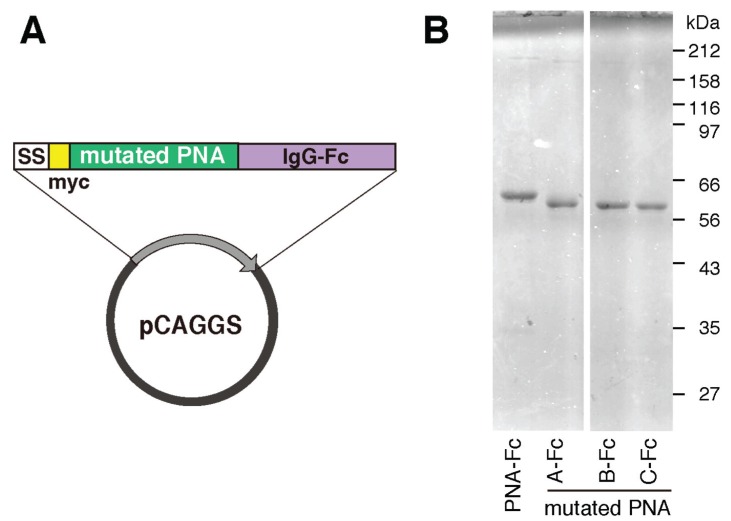
Plasmid construction and sodium dodecyl sulfate-polyacrylamide gel electrophoresis (SDS-PAGE) analyses of mutated PNA-Fc fusion protein. (**A**) The pCAGGS vector was used to express myc-tagged mutated PNA fused to Fc domain of human IgG; (**B**) SDS-PAGE analyses of purified wild-type and mutated (A–C) PNA-Fc proteins under reducing conditions.

**Figure 7:**
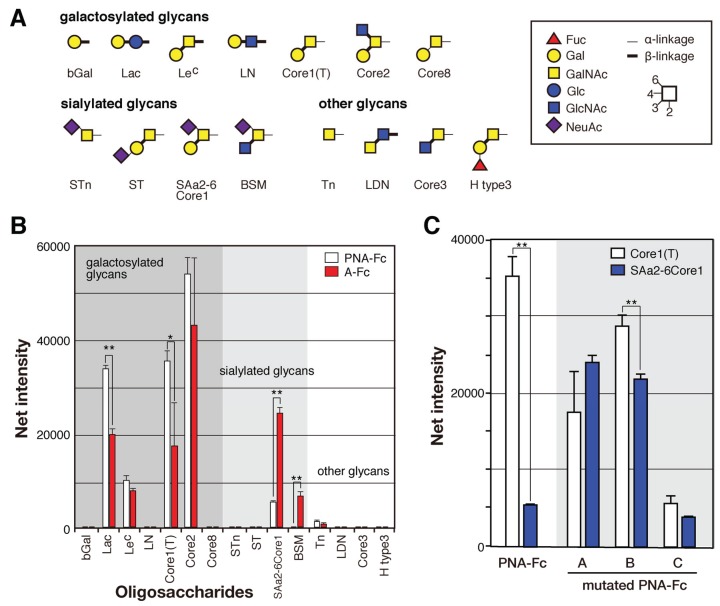
Binding of wild-type and mutated (**A**–**C**) PNA-Fc proteins to immobilized multivalent oligosaccharides and glycoproteins. (**A**) The carbohydrate structures of the polyacrylamide-based oligosaccharides and glycoproteins used for the glycan microarray analysis. All of the oligosaccharides and glycoproteins used in this experiment (98 in total) are shown in Figure A1. Symbols corresponding to each monosaccharide are shown in the inset panel. The thin and thick lines represent α- and β-linkages, respectively. The glycosidic linkage positions are shown by the numbers on the right side of the panel; (**B**) Glycan microarray analyses of the binding of wild-type PNA-Fc and A-Fc to the ligands shown in (**A**), as determined using an evanescent field-activated fluorescence scanner. The data are represented as the mean ± SD of n = 3 independent spots. ** *p* < 0.01; * *p* < 0.05 (Student’s *t*-test); (**C**) The binding of wild-type and mutated (A–C) PNA-Fc proteins to Galβ1-3GalNAcα-PAA (Core1(T)) and NeuNAcα2-6(Galβ1-3)GlcNAcα-PAA (SAa2-6Core1) as determined by glycan microarray analyses. The data are represented as the mean ± SD of n = 3 independent spots. The binding of wild-type and mutated PNAs against other glycans is shown in Figure A2. ** *p* < 0.01 (Student’s *t*-test).

**Figure A1:**
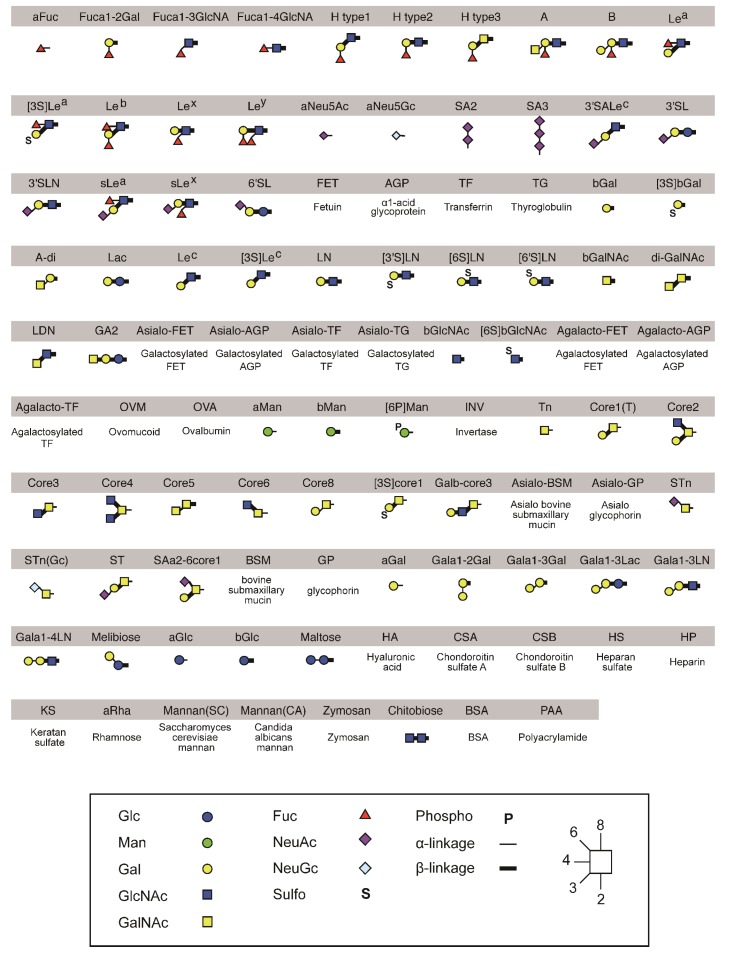
Structures of polyacrylamide-based oligosaccharides and glycoproteins used for the glycan microarray analysis.

To compare their sugar-binding specificities, each cDNA encoding recombinant wild-type and mutated PNA proteins (A to C) were fused to that of Fc region of human immunoglobulin (Figure 6A) and transformed into HEK293 cells. Each PNA-IgG Fc fusion protein (PNA-Fc) was purified from the culture medium of transformed cells using protein A-Sepharose columns (Figure 6B) and analyzed using a glycan microarray containing a variety of polyacrylamide-derived oligosaccharide polymers and glycoproteins (Figure A1). PNA is used widely as a Galβ1-3GalNAc (T antigen)-specific probe [20,21] because its ability to bind to this oligosaccharide is completely abrogated by sialylation of galactose and/or *N*-acetylgalactosamine [22,23]. Wild-type PNA-Fc bound to galactosylated glycans, namely, Galβ1-3GalNAcα-, Galβ1-3(GlcNAcβ1-6)GalNAcα-, Galβ1-4Glcβ-, and Galβ1-3GalNAcβ-PAA (Core1(T), Core2, Lac, and LeC in Figure 7A, respectively), but not to sialylated glycans. By contrast, the mutated PNA clone A Fc fusion protein (A-Fc), which was enriched by binding to NeuAcα2-6(Galβ1-3)GalNAcα-PAA (SAa2-6Core1, Figure 7), bound preferentially to the sialylated glycans NeuAcα2-6(Galβ1-3)GalNAcα-PAA and bovine submaxillary mucin (BSM, Figure 7), whereas its ability to bind to galactosylated glycans was lower than that of wild-type PNA-Fc (Figure 7B). Bovine submaxillary mucin is rich in sialylated *O*-glycans, NeuAcα2-6GalNAc (sialyl Tn (STn), Figure 7A), and NeuAcα2-6(GlcNAcβ1-3)GalNAc [24]; because A-Fc showed no affinity for STn and non-sialylated GlcNAcβ1-3GalNAc (Core3, Figure 7A), there may be the possibility that A-Fc can also bind to NeuAcα2-6(GlcNAcβ1-3)GalNAc. Furthermore, like wild-type PNA-Fc, A-Fc was also unable to bind to similar GalNAc- and Fuc-terminated sugar chains (other glycans, Figure 7B). Overall, these observations indicate that A-Fc acquired the ability to bind to sialylated NeuAcα2-6(Galβ1-3)GalNAc, but its affinity for desialylated ligands was attenuated compared to that of wild-type PNA (Figure 7C). To our knowledge, this study is the first report of a leguminous lectin that could bind stronger to sialylated NeuAcα2-6(Galβ1-3)GalNAc compared to asialo-Gal-GalNAc.

**Figure A2:**
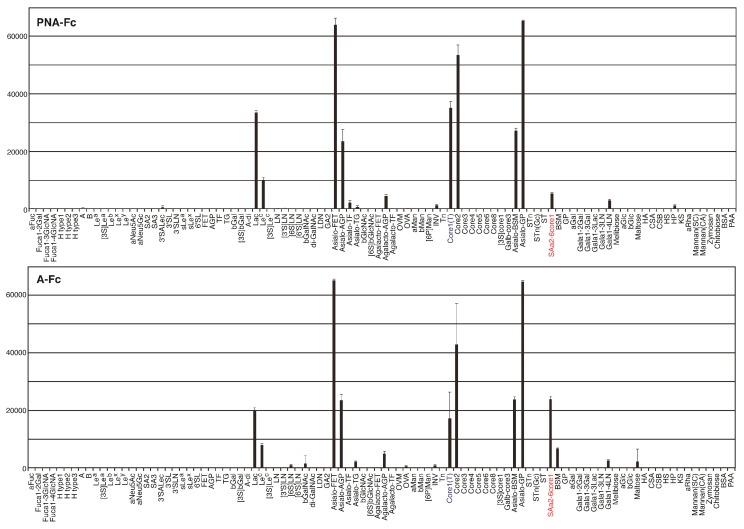

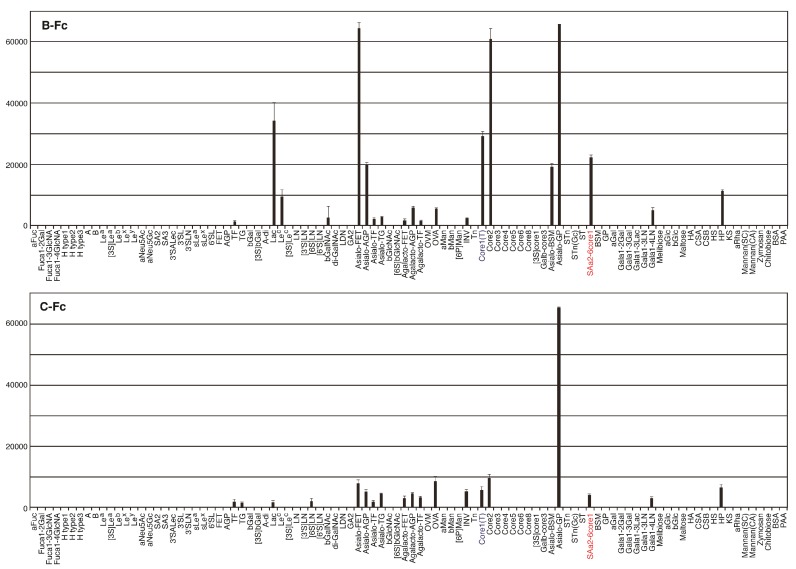
Glycan microarray analysis of wild-type and mutated PNA-IgG Fc fusion proteins.

Fc fusion proteins of the two other mutated PNA clones (B-Fc and C-Fc) were expressed, purified (Figure 7B), and subjected to a glycan microarray analysis as described above. B-Fc showed an enhanced affinity for sialylated glycans and its binding profile was similar to that of A-Fc. C-Fc bound to asialo-glycophorin A, which has 15 *O*-glycans of the structure Galβ1-3GalNAc (Sigma-Aldrich, St. Louis, MO, USA, partially sialylated). C-Fc also showed a little affinity for other glycans or glycoproteins including sialylated NeuAcα2-6(Galβ1-3)GalNAc (Figure A2), but its affinity for desialylated ligands was relatively attenuated compared with that of PNA-Fc (Figure 7C). Like PNA-Fc, mutated PNA-Fc proteins also showed high affinity for asialo-glycophorin A and Galβ1-3GalNAc (Core1(T), Figure 7A), suggesting that mutated clones with a PNA-like scaffold may share some common characteristics. Attempts to evaluate the sugar-binding specificities more precisely and determine the dissociation constant (*K*_d_) values of the mutated PNA-IgG Fc fusion proteins using frontal affinity chromatography and surface plasmon resonance (using BIAcore technology) were unsuccessful due to their weak binding to ligands.

### 2.5. The Amino Acid Sequences of Loops C and D from the Mutated PNA Proteins Isolated Using NeuAcα2-6(Galβ1-3)GalNAc

As mentioned above, in the screening performed using NeuAcα2-6(Galβ1-3)GalNAc as a probe, three different mutated PNA clones were generated. Initially, the randomized loop C and loop D lectin libraries were screened for mutant PNA proteins that had novel sugar-binding specificities and differed from wild-type PNA in both amino acid sequence and length. However, all of the mutated PNA clones obtained contained eight amino acids in loop C. Although the length of loop C differs among galactose-, mannose-, *N*-acetylglucosamine-, and *N*-acetylgalactosamine-binding leguminous lectins [17], the insertion of additional amino acid(s) into this region may disrupt the ability of ligands to access the carbohydrate recognition site. All of the mutated PNA clones enriched from the randomized loop C and loop D lectin libraries that retained sugar-binding activity contained mutations in loop C only, suggesting that the substitution and elongation of loop D abrogates the sugar-binding ability of PNA.

It is well known that an asparagine residue located in the middle of loop C is strictly conserved in leguminous lectins and the substitution of this residue causes a loss of activity [25,26,27]. Conservation of the asparagine residue in loop C is explained by its involvement in coordinate binding to calcium ions and cooperative hydrogen bonding to sugar ligands. To avoid disrupting this characteristic, we replaced the asparagine residue in loop C with glutamine, glutamic acid, or aspartic acid; however, all of the obtained mutated PNA clones retained asparagine at residue 127 in loop C (Figure 3C). This result demonstrates that conserved asparagine 127 in PNA is essential for its sugar-binding ability.

**Figure 8:**
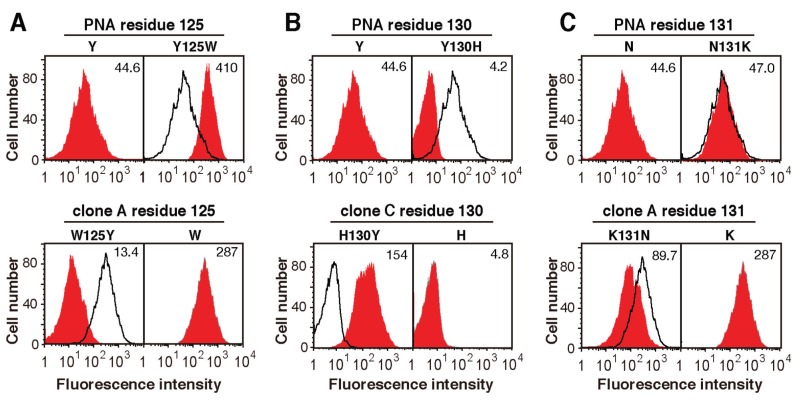
Flow cytometric analyses of the effects of single amino acid substitutions in loop C on the ability of wild-type and mutated PNAs to bind to HL60 cells. (**A**) The ability of wild-type PNA-Fc to bind to HL60 cells before (Y) and after Tyr125 was mutated to tryptophan (Y125W). Similarly, the binding of A-Fc to HL60 cells was measured before (W) and after Trp125 was replaced with tyrosine (W125Y); (**B**) The ability of wild-type PNA-Fc to bind to HL60 cells before (Y) and after Tyr130 was mutated to histidine (Y130H). Similarly, the binding of C-Fc to HL60 cells was measured before (H) and after His130 was replaced with tyrosine (H130Y); (**C**) The ability of wild-type PNA-Fc to bind to HL60 cells before (N) and after Asn131 was mutated to lysine (N131K). Similarly, the binding of A-Fc to HL60 cell was measured before (K) and after Lys131 was replaced with asparagine (K131N). The numbers in each panel indicate the mean fluorescence intensity. In the panels showing results for the PNA proteins in which residue 125, 130, or 131 was substituted, the solid lines and red histograms indicate binding before and after substitution, respectively. Similarly, in the panels showing results for mutated PNA-Fc proteins in which residue 125, 130, or 131 was substituted, the solid lines and red histograms indicate binding before and after substitution, respectively.

Residue 125 of the wild-type and mutated PNA proteins was tyrosine or tryptophan (Figure 3C). A previous X-ray crystallographic analysis of PNA complexed with its sugar ligand showed that Tyr125 of PNA interacts with sugar rings via van der Waals contacts. Similarly, tryptophan also seems to bind to sugar rings via van der Waals contacts. When Tyr125 of wild-type PNA was changed to tryptophan, the binding of PNA-Fc to HL60 cells, which expresses Galβ1-3GalNAc and NeuAcα2-6(Galβ1-3)GalNAc as major *O*-glycans on their cell surfaces [28], was increased by more than nine-fold (Figure 8A). Similarly, when Trp125 of mutated PNA clone A was replaced with tyrosine, its ability to bind to HL60 cells was decreased by approximately 20-fold (Figure 8A). Therefore, the sugar-binding ability of both PNA and mutated PNA was in the order of Trp125 > Tyr125.

Residue 130 of the wild-type and mutated PNA proteins was tyrosine, phenylalanine, or histidine (Figure 3C). An X-ray crystallographic analysis revealed that Tyr130 in wild-type PNA forms hydrogen bonds with structural water molecules [29]. When Tyr130 was changed to histidine, the binding of PNA-Fc to HL60 cells was decreased by 90%, whereas replacement of His130 in mutated PNA clone C with tyrosine increased its ability to bind to HL60 cells by more than 30-fold (Figure 8B).

In mutated PNA clone A, which had a stronger sugar-binding ability than the other mutated clones obtained and was identified by screening for binding to sialylated NeuAcα2-6(Galβ1-3)GalNAc, the asparagine residue at position 131 of wild-type PNA was substituted to lysine. Positively charged amino acids in loop C may contribute to binding to sialic acid residues of the glycan ligand; therefore, we determined the contribution of Lys131 of mutated PNA clone A to ligand binding by replacing it with asparagine, which is the corresponding residue in wild-type PNA. As expected, the binding of A-Fc to HL60 cells, which express high levels of αGalNAc:α2,6-sialyltransferase [28], was decreased by approximately 70% when Lys131 was replaced with asparagine; however, the substitution of Asn131 in wild-type PNA with lysine did not increase its binding ability to the cell (Figure 8C). Though the substitution of Asn131 to Lys of wild-type PNA might change the target glycan from asialo-Galβ1-3GalNAc to the sialylated one on HL60 cell, these results suggest that Lys131 may contribute to the binding of PNA to acidic glycans, but other amino acids in loop C may help to promote the interaction of Lys131 with sialic acid residues.

## 3. Discussion

Previously, we generated several mutated lectins with altered sugar-binding specificities using phage-display [30] and ribosome-display systems [31,32]. Although phage-display and ribosome-display are convenient screening methods for the selection and evolution of peptides or single-chain antibodies [33,34,35], they have some disadvantages when used for the selection of mutated lectins. First, high molecular weight proteins are not suitable for expression in these systems because the correct folding of recombinant proteins does not occur efficiently in *E. coli* cells or *in vitro*. Furthermore, in the case of phage-display systems, the efficiency of display of objective proteins on phage particles is quite low and the technique is limited to low molecular weight peptides. Second, both phage-display and ribosome-display methods require washing steps to remove unbound materials, which may lead to dissociation of the weak interactions between lectins and glycans (*K_a_* ~ 10^4^ M^−1^).

To overcome the difficulties associated with conventional methods of protein display, we successfully established a mammalian cell surface display (MCSD) method for the screening of mutated lectins having sugar-binding activity. Because mammalian cells have a sophisticated protein quality control system, which ensures that correctly folded proteins are expressed preferentially in the cell, the MCSD method enables the expression of high molecular weight proteins. Another advantage of the MCSD method is that weak sugar-protein interactions can be monitored by the transduced expression of GFP in the cell, and subsequent washing steps to remove unbound ligands do not affect this signal. Furthermore, because multivalency increases the avidity of the interaction, the MCSD method is able to monitor the weak binding of lectins to glycans; in fact, one of the mutated lectins obtained using the MCSD method showed almost undetectable affinity for many glycans or glycoproteins when tested using glycan microarrays (Figure A2, C-Fc). Based on the reasons described above, we propose that the MCSD method is superior to conventional methods in terms of efficiency, sensitivity, and reproducibility, not only for the screening of novel lectins but also for the identification of ligands of lectin-like receptors. A potential drawback of the MCSD system is the possibility of false negative GFP signals caused by the disruption of the binding of displayed lectins to immobilized glycans by endogenous glycans on the mammalian cell surface. To minimize this effect, it was necessary to control the cell culture conditions carefully, particularly the cell density and culture period.

It is well-known that PNA is specific for Galβ1-3GalNAc. By introducing amino acid substitutions into the sugar-binding loop C of PNA, we established novel mutated lectins that bound specifically to NeuAcα2-6(Galβ1-3)GalNAc. In a previous study, PNA was used to fractionate mouse or human thymocytes into two populations, namely PNA-agglutinated cells consisting mainly of immature cortical cells, and PNA-unagglutinated mature medullary thymocytes [36]. Leukocytes express leukosialin, a major *O*-glycan-containing sialoglycoprotein, and the structure of *O*-glycans depends on the leukocyte cell lineage and differentiation stage. NeuAcα2-6(NeuAcα2-3Galβ1-3)GalNAc is the major constituent of leukosialin-attached *O*-glycan in resting T-lymphocytes, whereas the level of Galβ1-4GlcNAcβ1-6(Galβ1-3)GalNAc is increased in activated T-lymphocytes [37]. *Maackia amurensis* hemagglutinin (MAH) binds specifically to the sialylated *O*-glycan NeuAcα2-6(NeuAcα2-3Galβ1-3)GalNAc but not NeuAcα2-6(Galβ1-3)GalNAc [38]. Since the *O*-glycan structures on leukocytes are useful markers of immune cell subpopulations along with MAH and wild-type PNA, mutated PNA clones A, B and C could be candidates for convenient cell probes.

The screening of several mutated PNAs with an affinity for NeuAcα2-6(Galβ1-3)GalNAc provided new insights into the amino acid residues that are responsible for the sugar-binding activity of leguminous lectins. In addition, mutated PNAs with no affinity for glycans were also obtained during the screening and the amino acid sequences of sugar-binding loops C and D of these proteins may also be helpful for understanding the involvement of critical amino acid residues in sugar-binding. First, we found that residue 127 of all of the mutated PNA proteins was asparagine; it is well known that asparagine residues in loop C are highly conserved across leguminous lectins, with the exception of the sialic acid-binding MAH lectins [17,39,40]. A previous X-ray crystallographic analysis of PNA demonstrated the involvement of asparagine in both coordinate binding to calcium ions and cooperative hydrogen bonding to sugar ligands. Second, residue 125 of wild-type and mutated PNAs was tyrosine or tryptophan. The residue at this position makes contact with a sugar ring via van der Waals interaction [29] and the mutation of Tyr125 in wild-type PNA to tryptophan-enhanced binding of the lectin to HL60 cells (Figure 8A). Similarly, the mutation of Trp125 of mutated PNA clone A to tyrosine decreased its ability to bind to HL60 cells (Figure 8A). Understanding the importance of the specific residue at position 125 may be useful for the generation of engineered leguminous lectins with enhanced binding ability. Third, we hypothesized that basic amino acids in loop C may enhance the binding of PNA to sialic acid-containing glycans. The replacement of Lys131 of mutated PNA clone A with asparagine, the residue found at this position in wild-type PNA, decreased its ability to bind to mammalian cells; however, the mutation of Asn131 in wild-type PNA to lysine did not affect its binding ability (Figure 8C). In sialic acid-binding *Maackia amurensis* lectins, a lysine residue in loop B forms hydrogen bonds with the carboxyl group of sialic acid in an unconventional binding mode [41]. Additional studies, such as X-ray crystallographic analyses, will clarify the involvement of these lysine residues in sugar recognition.

The length and composition of loop D of lectins are also thought to be involved in determining the sugar-binding specificity. However, although we prepared PNA proteins containing mutations in loop D, all of the mutated clones that retained sugar-binding ability contained the wild-type loop D sequence. Substitutions of a few amino acids in loop D might cause a conformational change in the sugar-binding site, resulting in a loss of sugar-binding ability.

Although PNA is specific for Galβ1-3GalNAc, this study raised the possibility that mutations in PNA produce novel lectins that are capable of recognizing Galβ1-3GlcNAc, Galα1-3Gal, GlcNAcβ1-3GalNAc, and Galα1-3GalNAc. Since efficient enrichment of the mutated PNAs was achieved using galactosylated glycans as ligands (Figure 4), it is possible that the scaffold of PNA can be optimized to enable binding of terminal galactose residues. The lengths of loop C from galactose/*N*-acetylgalactosamine-binding, mannose/glucose-binding, *N*-acetylglucosamine-binding, and sialic acid-binding leguminous lectins are approximately eight, eight, ten, and ten residues, respectively [17,40]. Based on a relationship between the length of loop C and its monosaccharide-binding specificity, we hypothesized that mutated PNAs specific for mannose, fucose, or *N*-acetylglucosamine could be established by adjusting the length of loop C (Figure 1D). However, the lengths of the loop C regions in the mutated PNA clones obtained using NeuAcα2-6(Galβ1-3)GalNAc were the same as that of wild-type PNA loop C. Furthermore, both the lengths and amino acid sequences of the loop D regions in the mutated PNA clones obtained using NeuAcα2-6(Galβ1-3)GalNAc as a ligand were the same as those of the wild-type PNA loop D. Other clones with different loop C and/or loop D lengths were also obtained during the screening; however, these clones did not show any sugar-binding ability in the reporter assay. Alterations in the length of loop C in the PNA scaffold may cause decreased expression of the protein, resulting in inefficient screening of mutated PNAs.

## 4. Experimental Section

### 4.1. Cells and Reagents

A human T cell hybridoma (2B4) harboring a GFP-reporter gene under the control of the nuclear factor of activated T cells (NF-AT) was kindly provided by Dr. H. Arase (Osaka University, Osaka, Japan) [42]. The retroviral vector, pMXs vector, and Plat-E retrovirus-packaging cells were provided by Dr. T. Kitamura (The Institute of Medical Sciences, The University of Tokyo, Tokyo, Japan) and were used for retrovirus transduction throughout this study [43]. The HEK293 cell was obtained from the Cell Resource Center for Biomedical Research (Tohoku University, Miyagi, Japan). The Plat-E cells were maintained in DMEM (Invitrogen, Carlsbad, CA, USA) supplemented with 25 mM HEPES (pH 7.4), 2 mM glutamine, 10 µg/mL blasticidin S (Invitrogen), and 1 µg/ml puromycin (Sigma-Aldrich). The 2B4 and HEK293 cells were cultured in DMEM supplemented with 10% heat-inactivated FBS, 2 mM glutamine, and 25 mM HEPES (pH 7.4). All cell lines were cultured at 37 °C under 5% CO_2_ conditions.

### 4.2. Establishment of PNA and Mutated PNA Library-Expressing Reporter Cells

Total RNAs were extracted from germinated peanut (*Arachis hypogaea*) seeds using the RNeasy Mini Kit (Qiagen, Valencia, CA, USA), and cDNAs were prepared by SuperScript III First-Strand Synthesis System (Qiagen). The cDNA encoding PNA was amplified by PCR using a forward primer (5'-CGGAATTCGCCGAAACAGTTTCCTTCC-3') and a reverse primer (5'-CCGCTCGAGTGCACTTGCACTTGCCATATTCAT-3') that contained an internal EcoRI and XhoI site, respectively. The obtained cDNA encoded a natural variant of PNA in which His139, Leu235, and Gly236 were substituted with Tyr, Arg, and Ala, respectively (AB917153) [44]. To express PNA on the surface of the 2B4 reporter cells, pMXs vectors containing cDNAs encoding the CD8β signal sequence, followed by myc-tagged PNA, the natural killer cell p46-related protein stalk domain, the CD8α transmembrane domain, and the mouse CD3ζ cytoplasmic domain (pMXs-PNA-CD3ζ), were constructed (Figure 1A). A PNA library containing mutations in loop C of the carbohydrate recognition domain was constructed by amplifying and ligating the 5' and 3' halves of the PNA cDNA (Figure 1C). The 5' half was amplified by PCR using the following primers: forward, 5'-GAAGGCCTCCCACTGATCACGTTGGA-3', and reverse, 5'-AACATTCTCGAGTGCACTTGCCATATTCAT-3'. The 3' half was also amplified by PCR and the region corresponding to loop C was randomized by using the following forward primer and degenerate reverse primers: forward, 5'-CGGAATTCGCCGAAACAGTTTCCTTCC-3'; reverse C1, 5'-ATCMNNMNNMNNMNNGTTMNNMNNMNNATCAAACTCCACTCCAAC-3'; reverse C2, 5'-ATCMNNMNNMNNMNNNTSMNNMNNMNNATCAAACTCCACTCCAAC-3'; reverse C3, 5'-ATCMNNMNNMNNMNNNTBMNNMNNMNNMNNATCAAACTCCACTCCAAC-3'; reverse C4, 5'-ATCMNNMNNMNNMNNNTBMNNMNNMNNMNNMNNATCAAACTCCACTCCAAC-3'; reverse C5, 5'-ATCMNNMNNMNNMNNNTBMNNMNNMNNMNNMNNMNNATCAAACTCCACTCCAAC-3'; or reverse C6, 5'-ATCMNNMNNMNNMNNNTBMNNMNNMNNMNNMNNMNNMNNATCAAACTCCACTCCAAC-3' (where M = A or C, N = A, T, G, or C, and B =T, G, or C). The amplified DNAs were inserted into pMXs-PNA-CD3ζ in place of the wild-type PNA cDNA to generate pMXs-PNALibC-CD3ζ. A loop D carbohydrate-recognition domain library of PNA was also constructed using a similar strategy to that described above. Briefly, the 5' half of the PNA cDNA was amplified by PCR using the following primers: forward, 5'-CGGAATTCGCCGAAACAGTTTCCTTCC-3', and reverse, 5'-AAGTTAACCGAACTTGACCCTCT-3'. The 3' half of the PNA cDNA containing loop D was also amplified by PCR using the following reverse primer and degenerate forward primers: reverse, 5'-CCCTTTTTCTGGAGACTAAAT-3'; forward D1, 5'-TTTCTGCCTCCGGCNNMNNMNNMNNMNNMNNMATACATCTCATCCGTTCA-3'; forward D2, 5'-TTTCTGCCTCCGGC NNMNNMNNMNNMNNMNNMNNMATACATCTCATCCGTTCA-3'; forward D3, 5'-TTTCTGCCTCCGGCNNMNNMNNMNNMNNMNNMNNMNNMATACATCTCATCCGTTCA-3'; or forward D4, 5'-TTTCTGCCTCCNNMNNMNNMNNMNNMNNMATACATCTCATCCGT TCA-3'. The 5' and 3' halves were then ligated into pMXs to generate pMXs-PNALibD-CD3ζ. Finally, a PNA library containing mutations in both loop C and loop D was constructed. Briefly, the cDNAs containing randomized loop C were obtained by digestion of the PNA loop C library with PacI and HindIII, and then inserted into pMXs-PNALibD-CD3ζ at the same sites to generate pMXs-PNALibCD-CD3ζ. The constructed plasmids were transformed into *E. coli* ElectroMAX DH10B cells (Invitrogen) by electroporation (1.8 kV, 186 ohm, and 50 μF for 7.8 ms). The plasmids (originating from 1.96 × 10^7^ cfu; Table 1) were then purified from the propagated *E. coli* cells. After recovery of retrovirus particles formed by the transfection of Plat-E cells with pMXs-PNALibC-CD3ζ, pMXs-PNALibD-CD3ζ, or pMXs-PNALibCD-CD3ζ using Lipofectamine 2000 reagent (Invitrogen), 2B4 cells (2.5 × 10^6^), which contained a GFP-reporter gene under the control of NF-AT, were infected with the retroviruses to induce the cell surface expression of wild-type or mutated PNA.

### 4.3. Screening of Engineered Lectins from Mutated PNA Library-Expressing Cells by a GFP-Reporter Assay

The 2B4 cells expressing mutated PNA on their cell surface were stained with an anti-myc antibody (9E10; American Type Culture Collection, Manassas, VA, USA) and *R*-phycoerythrin-labeled goat anti-mouse IgG-F(ab’)_2_ (Beckman Coulter, Fullerton, CA, USA), and then enriched using a FACS Vantage SE cell sorter (BD Biosciences, San Jose, CA, USA) based on the expression of the myc-tag. The enriched 2B4 cells expressing mutated PNA were cultured in 6-well ELISA plates (Iwaki, Tokyo, Japan) coated with acrylamide-based multivalent sugar polymers (sugar-PAA) (GlycoTech, Gaitheresburg, MD, USA) for 16 h at 37 °C. The 2B4 cells with higher GFP expression (less than 3% of the population) were collected using the FACS Vantage SE instrument (BD Biosystems, San Jose, CA, USA) and cultured for the next round of sorting. After enrichment of the GFP-positive cells was repeated two or three times, each cell was cloned by limiting dilution. A reporter assay of each cloned cell grown on various sugar-PAA-coated wells was performed using the FACS Calibur system (BD Biosciences), and the data were analyzed using FlowJo software (TreeStar, San Carlos, CA, USA).

Alternatively, GFP-positive 2B4 cells expressing mutated PNA, which had been cultured in a 9 mm petridish (BD Falcon, Bedford, MA, USA) coated with acrylamide-based multivalent sugar polymers, were visualized by fluorescence microscopy and approximately twenty GFP-positive cells were collected using the piezo micromanupilator, PMM-150HJ (Prime tech, Ibaraki, Japan).

### 4.4. Isolation and Sequencing of Cloned Mutated PNA cDNAs

Genomic DNA was extracted from the cloned GFP-positive cells using the FlexiGene DNA Kit (Qiagen) according to the manufacturer’s protocol. The cDNAs encoding mutated lectins were then amplified by PCR using a forward primer (5'-CGGAATTCGCCGAAACAGTTTCCTTCC-3') containing an *Eco*RI site and a reverse primer (5'-CCGCTCGAGTGCACTTGCCATATTCAT-3') containing an *Xho*I site, subcloned into the pBluescript II SK(+) vector, and sequenced using an ABI 3500/3500xL genetic analyzer (Applied Biosystems, Foster City, CA, USA). Each putative lectin cDNA was subcloned into pMXs-CD3ζ and expressed in 2B4 cells. Cloned lectin mutants expressed on the cell surface were subjected to GFP-reporter assays in several different sugar-PAA coated wells, as described above.

### 4.5. Preparation of Mutated PNA-IgG Fc Fusion Proteins

A cloned lectin cDNA fused to the cDNA encoding the Fc segment of human IgG_1_ (provided by Dr. H. Arase, Osaka University) was inserted into the pCAGGS vector to generate pCAGGS-mPNA-Fc [45]. HEK293 cells were transfected with pCAGGS-mPNA-Fc using Lipofectamine 2000 reagent, and cells stably secreting mutated PNA-Fc proteins were selected by culturing in medium containing 1 mg/mL G418 (Sigma-Aldrich). After the cloning of G418-resistant cells by limiting dilution, the clone that displayed the highest production of the mutated PNA-Fc fusion protein was chosen and subjected to a large-scale culture. The mutated PNA-Fc fusion protein was purified from conditioned media of the cells by affinity chromatography using a HiTrap rProtein A FF column (GE Healthcare, Piscataway, NJ, USA) and the AKTA Explorer system (GE Healthcare). The purity was confirmed by SDS-PAGE under reducing conditions. To introduce a single mutation into the cDNAs encoding PNA-Fc and mutated PNA-Fc clones A and D, the KOD-Plus-Mutagenesis Kit (Toyobo, Osaka, Japan) was used according to the manufacturer’s protocol.

### 4.6. Glycan Microarray

The sugar-binding specificities of the mutated PNA proteins were analyzed using a glycan microarray (version 4.2) [46]. Briefly, 100 µL of mutated PNA-Fc fusion protein (50 µg/mL) was loaded onto the glycan microarray and incubated at 20 °C for 18 h. After washing with probing buffer (10 mM Tris-HCl (pH 7.4) containing 0.15 M NaCl and 0.02% Tween 20), 100 µL of Cy3-labeled donkey anti-human IgG-Fcγ antibody (1 µg/mL; Jackson ImmunoResearch, West Grove, PA, USA) in probing buffer was applied to the microarray and incubated at 20 °C for 3 h. After a further wash with probing buffer, the binding of mutated PNA-Fc fusion protein was detected using the SC-profiler evanescent field fluorescence-assisted scanner (GP Biosciences Ltd., Yokohama, Japan) in the Cy3 mode.

## 5. Conclusions

We established a method for the efficient expression of leguminous lectin subunits on the surface of mammalian cells without the loss of sugar-binding ability. To evaluate the amino acid sequence, especially in its sugar-binding loops, and the sugar-binding specificity of lectins, we introduced random mutations in the sugar-binding loops of peanut agglutinin, expressed on the surface of mammalian cells, and successfully screened for mutated PNAs with novel sugar-binding specificities. The data demonstrated that sugar-binding loop C was largely involved in sugar-binding specificity of the lectin and critical amino acid residues associated with sugar-binding activity and specificity were clarified.
